# The Narrow Footprint of Ancient Balancing Selection Revealed by Heterokaryon Incompatibility Genes in *Aspergillus fumigatus*

**DOI:** 10.1093/molbev/msae079

**Published:** 2024-04-23

**Authors:** Ben Auxier, Jianhua Zhang, Francisca Reyes Marquez, Kira Senden, Joost van den Heuvel, Duur K Aanen, Eveline Snelders, Alfons J M Debets

**Affiliations:** Laboratory of Genetics, Wageningen University & Research, Wageningen, the Netherlands; Laboratory of Genetics, Wageningen University & Research, Wageningen, the Netherlands; Laboratory of Genetics, Wageningen University & Research, Wageningen, the Netherlands; Laboratory of Genetics, Wageningen University & Research, Wageningen, the Netherlands; Laboratory of Genetics, Wageningen University & Research, Wageningen, the Netherlands; Laboratory of Genetics, Wageningen University & Research, Wageningen, the Netherlands; Laboratory of Genetics, Wageningen University & Research, Wageningen, the Netherlands; Laboratory of Genetics, Wageningen University & Research, Wageningen, the Netherlands

**Keywords:** allorecognition, cell death, balancing selection

## Abstract

In fungi, fusion between individuals leads to localized cell death, a phenomenon termed heterokaryon incompatibility. Generally, the genes responsible for this incompatibility are observed to be under balancing selection resulting from negative frequency-dependent selection. Here, we assess this phenomenon in *Aspergillus fumigatus*, a human pathogenic fungus with a very low level of linkage disequilibrium as well as an extremely high crossover rate. Using complementation of auxotrophic mutations as an assay for hyphal compatibility, we screened sexual progeny for compatibility to identify genes involved in this process, called *het* genes. In total, 5/148 (3.4%) offspring were compatible with a parent and 166/2,142 (7.7%) sibling pairs were compatible, consistent with several segregating incompatibility loci. Genetic mapping identified five loci, four of which could be fine mapped to individual genes, of which we tested three through heterologous expression, confirming their causal relationship. Consistent with long-term balancing selection, trans-species polymorphisms were apparent across several sister species, as well as equal allele frequencies within *A. fumigatus*. Surprisingly, a sliding window genome-wide population-level analysis of an independent dataset did not show increased Tajima's *D* near these loci, in contrast to what is often found surrounding loci under balancing selection. Using available de novo assemblies, we show that these balanced polymorphisms are restricted to several hundred base pairs flanking the coding sequence. In addition to identifying the first *het* genes in an *Aspergillus* species, this work highlights the interaction of long-term balancing selection with rapid linkage disequilibrium decay.

## Introduction

The ability to differentiate self from nonself is a ubiquitous factor of multicellular life. Best studied in mammalian major histocompatibility loci, this process prevents fusion between genetically distinct organisms, in effect delimiting an individual (Billingham et al. 1953; Boehm 2006). Distinguishing self from nonself is also important for fungi, as the fungal mycelium (the threadlike structure of filamentous fungi) benefits from fusions within its own mycelial network (Bastiaans et al. 2015). However, fusion events between mycelia of different individuals allow for the propagation of selfish genetic elements such as viruses (Anagnostakis 1983), plasmids and mitochondria (Debets et al. 1994), and nuclei (Bastiaans et al. 2016; Grum-Grzhimaylo et al. 2021). To discriminate between self and nonself fusions, fungal species possess a robust multigenic incompatibility system based on the alleles of *het* genes, which trigger cell death when gene products from two alleles are found in the same cytoplasm (Glass and Dementhon 2006). Within a fungal population, these *het* genes are highly polymorphic and segregation of alleles from multiple unlinked genes results in two randomly selected fungal individuals being virtually guaranteed to have a distinct combination of *het-*gene alleles (Nauta and Hoekstra 1994; Czárán et al. 2014; Gonçalves and Glass 2020).

A common observation of such *het* genes is that they appear to be under balancing selection. First shown in *het*-c of *Neurospora crassa*, this balancing selection results in alleles being found in even frequencies in the population (i.e. two alleles found at a 1:1 ratio, or three alleles at 1:1:1) (Wu et al. 1998). This pattern of even allele frequencies appears to be quite general, being found in many fungal species (Bastiaans et al. 2014; Milgroom et al. 2018; Ament-Velásquez et al. 2022). While it can be difficult to disentangle the precise cause of balancing selection (Spurgin and Richardson 2010), the generally accepted cause for nonself recognition genes such as *het* genes is negative frequency-dependent selection (Nauta and Hoekstra 1994; Muirhead et al. 2002). Under this assumption, the fitness of a *het-*gene allele is based on the presence of alternate alleles. Individuals with an allele that is common in a population are less effective in detecting nonself fusions, since it is more likely that pairs of individuals will share this allele. Conversely, individuals with a rare allele will be able to effectively detect fusions with other individuals. This dynamic based on allele frequency prevents either allele from reaching fixation, and both alleles are maintained longer than expected by random processes, with alleles found closer related between rather than within a species (Charlesworth 2006). Such patterns have been found in all genera studied to date; *Neurospora*, *Cryphonectria*, and *Podospora* (Wu et al. 1998; Milgroom et al. 2018; Ament-Velásquez et al. 2022).

The fusion between two fungal individuals, which generally differ at multiple *het* loci, leads to the death of the fused cell (Garnjobst and Wilson 1956; Glass and Kaneko 2003). Some known *het* genes trigger death due to protein–protein interaction encoded by different alleles of the same genes (allelic), while other *het-*gene reactions are based on the protein products of alleles of separate tightly linked genes (nonallelic) within a larger haplotype (Kaneko et al. 2006). Some *het* genes utilize systems based on proteins similar to nucleotide-binding domain and leucine-rich repeat (NLRs), with a tripartite system of a recognition domain, a central nucleotide-binding domain, and a terminal protein-interaction domain, with strong parallels to the immune system of animals and plants (Uehling et al. 2017; Heller et al. 2018). The action of *het* genes can also be based on prion formation, converting the alternate protein product into a cytotoxic protein that destabilizes the plasma membrane (Debets et al. 2012; Saupe and Daskalov 2012). Recently, the involvement of gasdermin or gasdermin-like proteases has been recognized in several fungal lineages (Clavé et al. 2022). Still, for many *het* genes the mode of action remains unknown. In the *Sordariomycete* genera *Neurospora* and *Podospora* many *het* genes contain a protein domain of unknown function, termed the HET domain (Saupe and Glass 1997; Zhao et al. 2015). While this domain is found in other fungi, the relationship between the HET domain and functional *het* genes in other fungal lineages remains uncertain (Fedorova et al. 2008; Dyrka et al. 2014; van der Nest et al. 2014).

The fungal species *Aspergillus fumigatus* is globally distributed and primarily decays dead plant material. Its spores are inhaled daily, and generally present no concern to the health of the organism. However, when the immune system is suppressed, such as during chemotherapy, immune-suppressive therapy following organ transplantation or following infection with influenza or SARS-CoV-2 in intensive care units, this fungus can invasively grow into the lung tissue, leading to a condition called invasive aspergillosis (Schwartz et al. 2020; Arastehfar et al. 2021; Arné et al. 2021; Chong and Neu 2021). Additionally, *A. fumigatus* can also chronically colonize human lungs in cystic fibrosis or chronic obstructive pulmonary disease patients, and the same clonal isolate can be recovered from a patient sequentially for years (Bhargava et al. 1989; de Valk et al. 2009). First-choice treatment for severe *Aspergillus* disease is the azole antifungal class targeting the fungal ergosterol pathways. Increasingly, antifungal resistance is found due to environmental exposure of the isolates prior to infection of a human host (Verweij et al. 2009). As a potential alternative to traditional drug targets, the manipulation of cell-death processes has been proposed to manage fungal infections (Weaver 2013; Hardwick 2018; Kulkarni et al. 2019). The first step for such research would be the identification of the genes that trigger heterokaryon incompatibility, the *het* genes, in *A. fumigatus*.

The genetics of *het* genes in other *Aspergilli* has been investigated previously, although not at a molecular level. Experimental studies in *A. nidulans* and *A. heterothallicus* both showed that incompatibility segregated in the progeny of sexual crosses, and was due to the action of several loci, although this was before the genomic era and specific genes were not identified (Kwon and Raper 1967; Anwar et al. 1993; Dales et al. 1993; Coenen et al. 1994). Using bioinformatic tools, sequence divergence combined with gene function information has been used as criteria to identify putative *het* loci in *A. fumigatus* (Fedorova et al. 2008). These diverged regions were termed “islands” of diversity in the *A. fumigatus* genome, with a highly divergent region flanked by large stretches of almost identical sequence. More recently, a reverse genetics study of genes encoding a HET domain in *Aspergillus oryzae* showed that the expression of alternate alleles of most genes with a HET domain did not produce an incompatibility reaction, although some led to a modest reduction in growth (Mori et al. 2019). Within *A. fumigatus* it appears that both field and clinical isolates are not capable of forming stable heterokaryons, indicating abundant presence of heterokaryon incompatibility genes (Weaver 2013; Zhang et al. 2019). Currently, *het* genes remain unidentified not only in *A. fumigatus* but in the genus *Aspergillus* in general.

Here, we combine whole-genome sequence data of sexual progeny from two heterokaryon-incompatible strains with phenotypic interactions to map the (in)ability to complement two auxotrophic mutations and form stable heterokaryons. Our presumption is that the inability to complement is related to *het-*gene allelic differences. We then investigate the putative function and evolutionary history of these loci across closely related species and finally, as we find the region affected by balancing selection is quite narrow, we consider what factors of the biology of *A. fumigatus* may affect the long-term genetic outcome of balancing selection.

## Results

### Heterokaryon Compatibility Testing

To map the genes causing incompatibility between our two parental strains, we phenotyped based on complementation of auxotrophic nitrate pathway mutations. In this method, two haploid strains with different nitrate-deficiency mutations (e.g. one *nir* and the other *cnx*) will show vigorous heterokaryotic growth on nitrate as a sole N-source from sustained cell fusion when compatible, but not when incompatible (supplementary fig. S1, Supplementary Material online). We produced from each of the two parents (AfIR974 and AfIR964) three nitrate deficient strains; a *nir*, a *cnx*, and a *nia* mutant. We then induced a sexual cross between the AfIR974 *cnx* and the AfIR964 *nir* mutants. Among the 193 offspring, the distribution of genotypes (42 *nir*; 51 *cnx*; 55 *nir*/*cnx*; 45 wt) was not significantly different from the expected 1:1:1:1 Mendelian ratio for the two segregating loci (*χ*^2^ test, *P* = 0.56).

To assess heterokaryon compatibility, we tested offspring against parents, as well as offspring against offspring, and a schematic can be found in supplementary fig. S1, Supplementary Material online. In total, there were 148 offspring that could be tested for heterokaryon compatibility to the *nia* mutant version of the parents (42 *nir* offspring, 51 *cnx* and 55 *nir/cnx*) (supplementary Data: table S1, Supplementary Material online). From these pairings, we recovered five successful combinations forming a heterokaryon (3.4%) after 3 days of incubation. Three offspring (A118, A189, and A108) could form heterokaryons with the parent AfIR964 *nia*, and two offspring (A166 and A142) were compatible with the parental AfIR974 *nia*. Offspring that were compatible with a parent were only compatible with one parent. To increase the number of compatible pairs recovered, we then tested each of the 42 *nir* strains against each of the 51 *cnx* strains of the offspring, resulting in testing of 2,142 sibling pairings. Of these, 166 pairs (7.7%) formed heterokaryons. Of these 166 pairs, visible heterokaryons developed within 3 days for 133 pairs, whereas for 33 pairs, heterokaryons were visible only after 5 days.

### Genetic Mapping

**Fig. 1.**
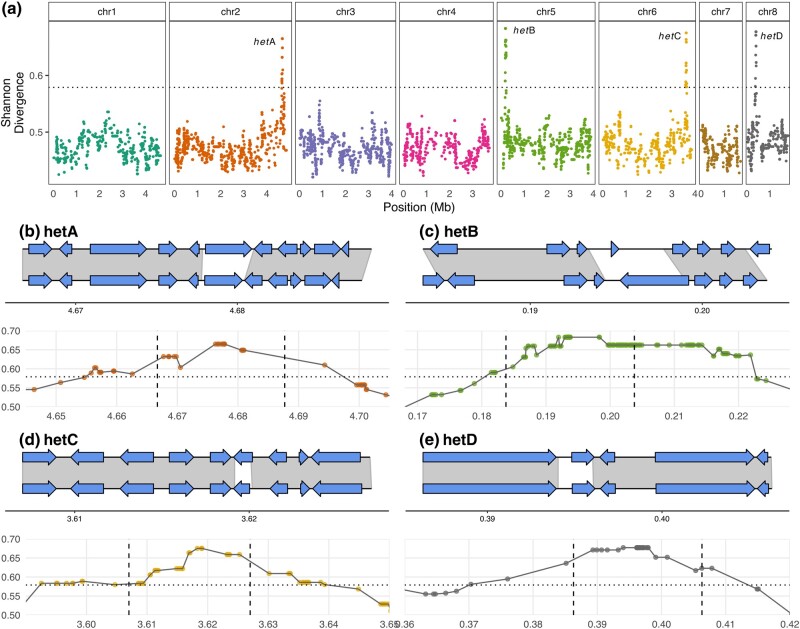
Mapping of *A. fumigatus het* loci based on heterokaryon-forming pairs. a) Genome-wide mapping of heterokaryon compatibility after 5 days of incubation. Dots indicate variant positions and associated Shannon Entropy phenotypic association. Significance threshold is determined by the top scoring 5% of 1,000 random phenotypic permutations. b–e) Fine mapping of *hetA/B/C/D* loci. The lower part of each figure shows a close-up of the locus in a) that rises above the significance threshold. Dashed vertical lines indicate the variable genomic region with gene models shown with AfIR964 above AfIR974. Arrows indicate predicted gene models and shading indicates regions with high sequence similarity between the two parental genomes. In these four loci, there is a region in the middle of the locus, with one associated gene, that is highly differentiated between parents, and lacks shading.

From the 166 heterokaryon-compatible sibling pairings and the 5 compatible parental–offspring pairs, we could form 15 vegetative compatibility groups (VCGs) (supplementary Data: table S2, Supplementary Material online). Using the assumption that VCGs have identical alleles at all *het* genes, we compared the genomes of the strains in each VCG at 14,153 variant sites previously identified (Auxier et al. 2023). As genotypes are expected to be similar within, but not between, groups we used the Shannon's Entropy metric to determine similarity (see Materials and Methods). Mapping by using the 166 isolates resulted in peaks on chromosomes 2, 5, 6, and 8, which we termed *hetA*, *hetB*, *hetC*, and *hetD*, respectively (Fig. 1a). Each of these peaks rose above the significance threshold of 0.567, the highest 5% of the permutations. For each of these peaks, the window of significant markers spanned approximately 40 kb with up to 20 predicted protein-coding genes (Fig. 1b–e). Within the larger VCGs, we observed a polymorphism where some pairings formed heterokaryons within 3 days, while other pairings formed heterokaryons after only 5 days (supplementary Data: table S2, Supplementary Material online). To investigate the genetic basis of this, we split the VCGs into sub-VCGs (i.e. we split VCG 1 into VCG 1a and 1b based on heterokaryon formation within 3 days) and performed a similar Shannon Entropy analysis (supplementary fig. S2 and supplementary Data: table S3, Supplementary Material online). This analysis recovered a single region on Chromosome 6, which we termed *hetE*.

For each of the four *hetA/B/C/D* loci, the sequence similarity was high across the locus between the parents (gray shading Fig. 1b–e) except near the center of the locus with a single coding region with low sequence similarity. For *hetA/C/D* this central divergent gene had two similarly sized alleles. For *hetB* we instead found that the two alleles had greatly differing coding sequence lengths, and in opposite directions. The *hetE* region was larger than the others, spanning approximately 80 kb, with ∼40 predicted protein-coding genes. The *hetE* locus contained three coding sequences in the AfIR974 haplotype and four coding sequences in the AfIR964 haplotype, all of which had low sequence similarity between the parents (supplementary fig. S2, Supplementary Material online).

To verify that the five loci identified represented all the *het* loci segregating between these two parents, we backcrossed offspring predicted to differ at only one identified *het* locus from a parent. We then scored 40 offspring from each of 9 such crosses, 2 independent crosses for *hetA/B/C/D* and 1 for *hetE*, for compatibility to the parental strain. In all crosses segregation of compatibility to the parent was not significantly different from a 1:1 ratio (supplementary Data: table S4, Supplementary Material online).

### *het* Gene Identification and Validation

**Fig. 2.**
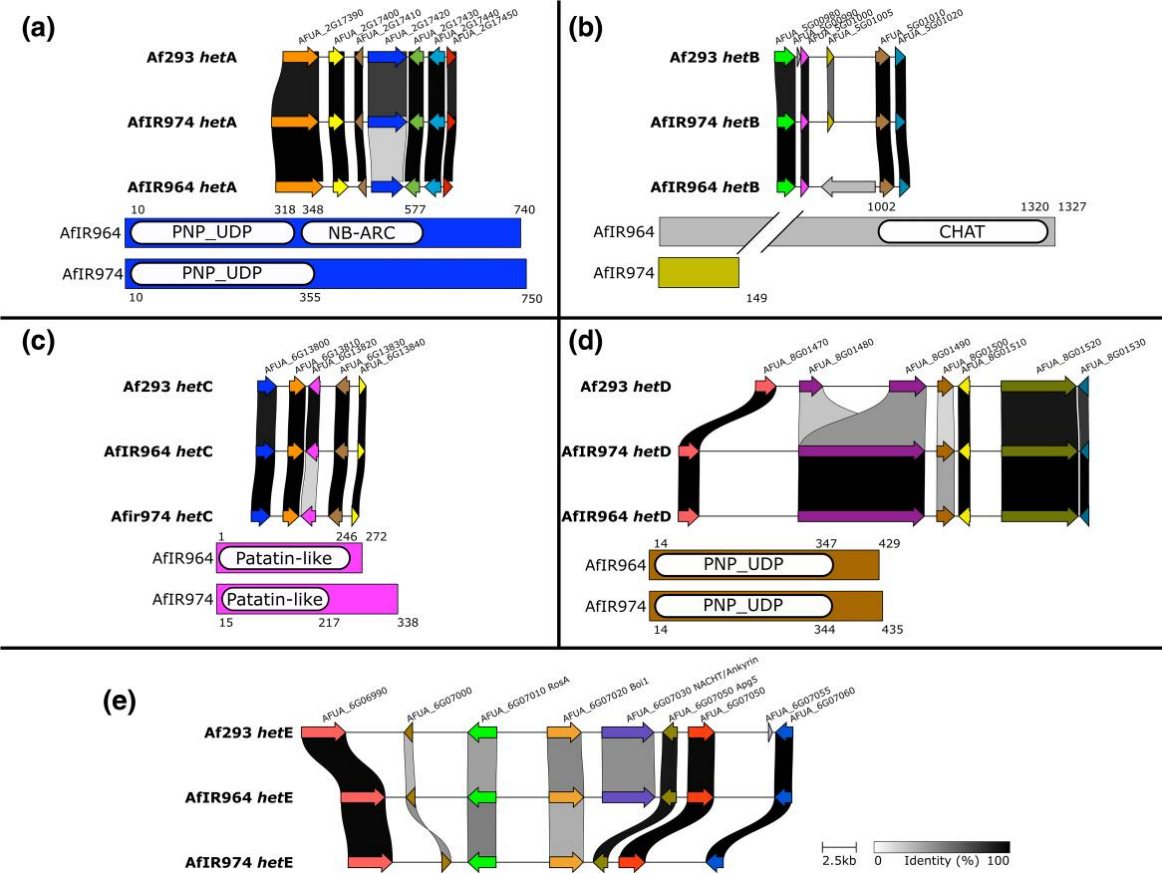
Genomic details of *het* genes. a–e) Top shows syntenic region of *het* gene with adjacent genes for AfIR974, AfIR964, and the reference strain Af293. Colors indicate gene identity, and links between genes indicate amino acid sequence similarity (legend in e). Bottom shows the protein sequence for each allele, with domains as predicted by InterPro. Colors of proteins reflect synteny analysis above.

The predicted protein products of the *hetA* alleles both contained a nucleotide phosphorylase PNP_UDP, as well as a predicted nucleotide-binding NB-ARC domain for the AfIR964 allele (Fig. 2a). The *hetB* gene has two idiomorphic alleles, with the AfIR964 allele predicted to encode a 1,327 residue protein that was predicted to contain a Caspase HetF Associated with Tprs (CHAT) domain, while the alternate *hetB* allele from AfIR974 was a similar nucleotide length but only a 149 residue protein was predicted, with no predicted functional domains (Fig. 2b). The alleles of the *hetC* gene were both predicted to produce a patatin-like protein (PLP) of similar lengths (Fig. 2c). The *hetD* alleles were also both predicted to encode similar-sized proteins, both with a single PNP_UDP phosphorylase domain (Fig. 2d). In contrast to the other *het* genes, the *hetE* locus contained multiple candidate genes all of which were polymorphic between parents (Fig. 2e). Of these, one gene lacked identifiable domains, while another was the orthologue of a *A. nidulans* characterized gene *rosA* (see Discussion) and one is a predicted homolog of yeast *boi1* (Fig. 2e). The final candidate gene in the *hetE* locus contained a NACHT domain as well as Ankyrin-repeats and was found in the AfIR964 allele and not present in the AfIR974 allele. Comparison with Af293 showed an alternate allele of the NACHT/Ankyrin gene with low sequence similarity (Fig. 2e).

**Fig. 3.**
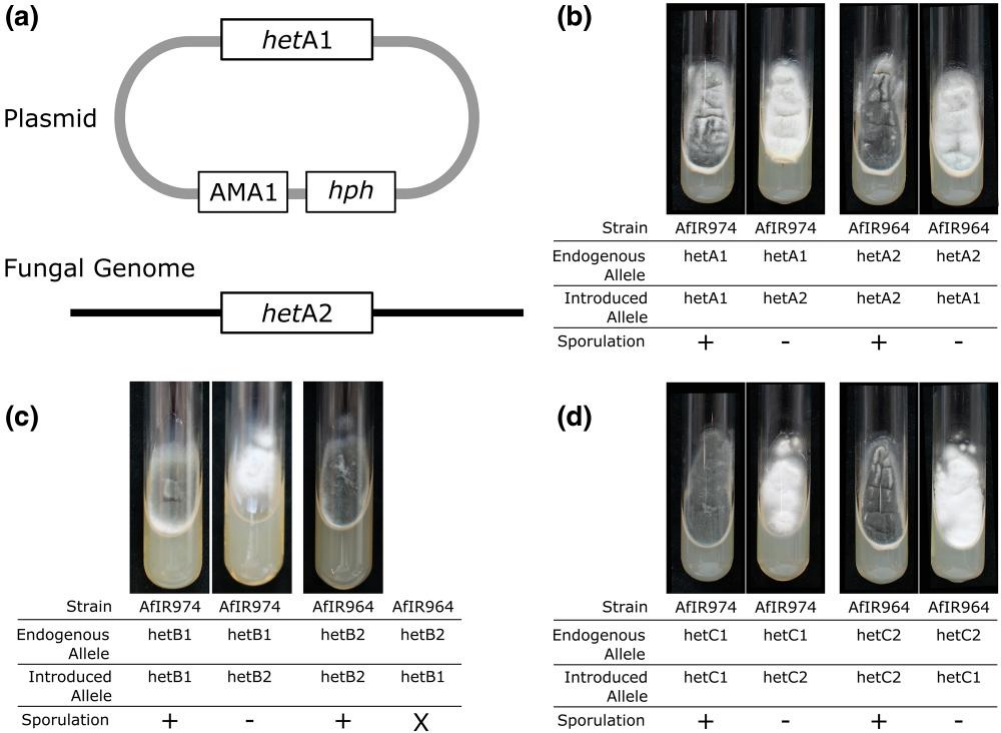
Validation of predicted *A. fumigatus het* genes using heterologous expression. a) Schematic drawing of introducing an alternate *het* allele by transformation with the nonintegrative AMA1 plasmid. b) Phenotypes of *hetA* transformants growing on hygromycin selective media, with transformants with endogenous alleles showing wild-type green sporulation, and transformants with the alternate allele with visible white mycelia due to lack of sporulation. c) Phenotypes as in b, but with transformants for *hetB* alleles. Note that AfIR964 with *hetB1* was not viable. d) Similar to b, but with transformants for *hetC* alleles.

We confirmed that the candidate *het* genes were causal to the phenotype using heterologous expression. Using a nuclear-localized autonomously replicating plasmid, we cloned each allele including ∼500 bp of flanking sequence for *hetA*, *hetB*, and *hetC* (Fig. 3a). We did not attempt validation of either *hetD* or any of the genes in the *hetE* locus. For each of the three loci tested, expression of the resident allele on the replicating plasmid led to no change in phenotype. However, introduction of the alternate allele led to an aberrant phenotype of abundant white mycelium and a complete absence of sporulation (Fig. 3b–d). For both *hetA* and *hetC* both alleles had equal effect, with the same white mycelial phenotype. For *hetB*, expression of the *hetB2* allele in AfIR974 produced this same white nonsporulating phenotype, but the expression of *hetB1* in AfIR964 resulted in initial nonsporulating colonies that were not viable after transfer on selective media. Transfer of colonies to media without selection pressure for maintenance of the replicating plasmid allowed rapid sectoring, restoring the wild-type green sporulation, indicating the phenotype resulted from the presence of the plasmid with the antagonistic *het* allele and not the transformation procedure (data not shown).

### Balancing Selection

**Fig. 4.**
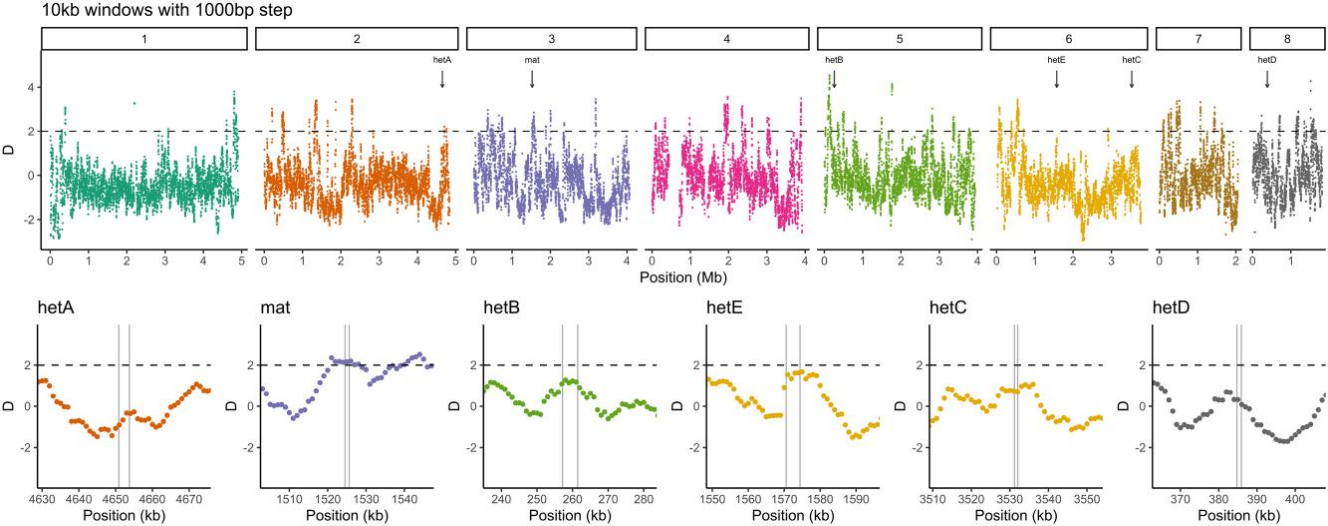
Tajima's *D* is not increased near *het* genes, nor near the mating-type locus. Top row shows the genome-wide *D* values calculated in 10 kb windows with a 1 kb step size. Chromosomes are separated by color, and the five *het* genes are indicated by arrows as well as the mating-type locus. The bottom row shows a closer view of each of the six loci.

As heterokaryon incompatibility genes are expected to be under balancing selection, we assessed the strength of this selection across *A. fumigatus* in two datasets. Using genome-wide variants of 213 UK samples, we calculated Tajima's *D* values in sliding windows of 10 kb (Fig. 4). This dataset had a mean *D* value of 0.05. Using window sizes of 10 kb, we did not find increased Tajima's *D* associated with any of our identified *het* genes nor with the mating-type locus (Fig. 4), neither when analyzed with other window sizes (supplementary figs. S3 and S4, Supplementary Material online). The nearest of our identified *het* genes to a high Tajima's *D* value was *hetB* on chromosome 5, although this high *D* value is near the start of the chromosome, and close inspection of the window surrounding *hetB* shows a *D* value below 2.

**Fig. 5.**
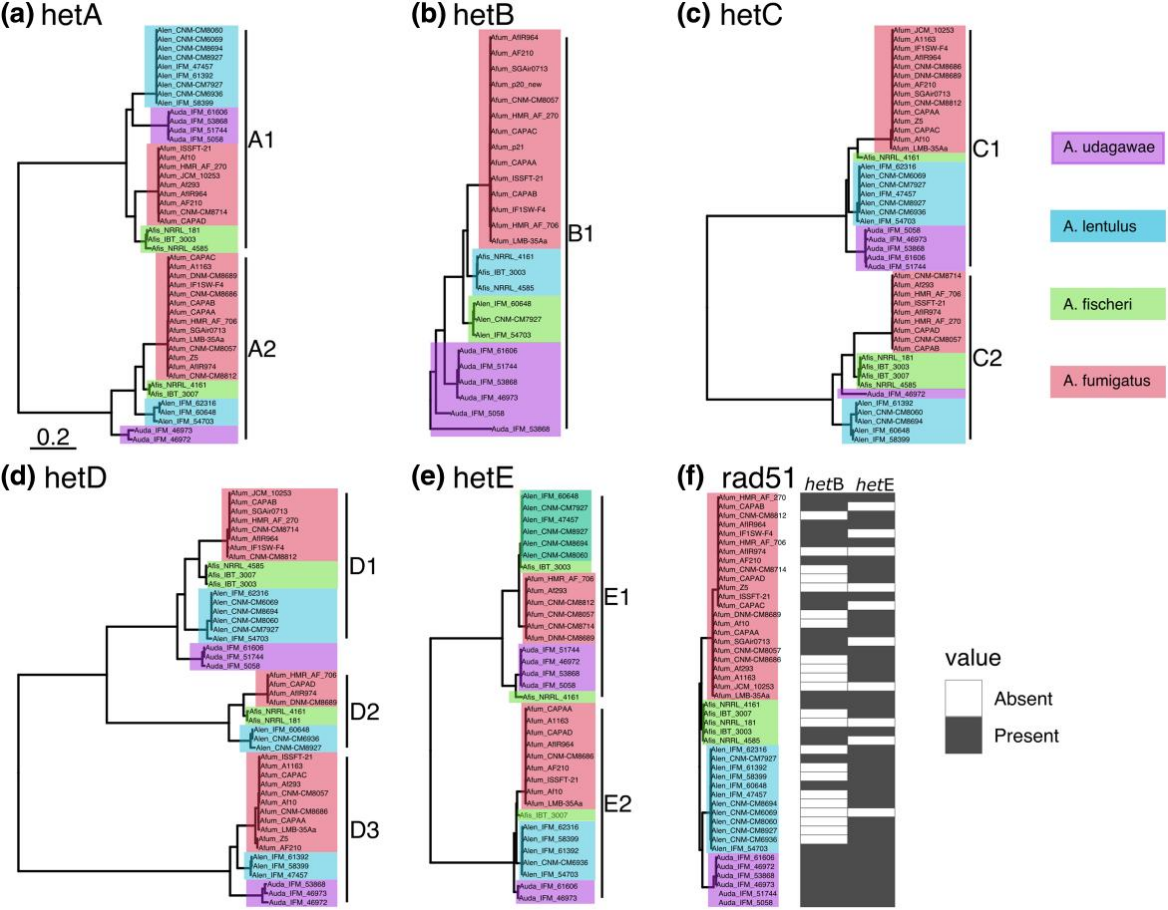
Phylogeny of predicted *het-*protein sequences shows trans-species polymorphisms. Maximum-likelihood unrooted phylogeny of amino acid sequence of related *Aspergilli*. Branch lengths indicate amino acid change, with tips colored according to species. Salmon indicates *A*. *fumigatus*, green *A*. *fischeri*, light blue *A*. *lentulus*, and purple *A*. *udagawae*. Note that b) and e) only include the alleles with a protein present and the absence alleles are indicated in f).

As an alternate measure of balancing selection on these *het* genes, we reconstructed the phylogenetic relationships between amino acid sequences of *het* genes from *A. fumigatus* as well as the closely related *A. fischeri*, *A. lentulus*, and *A. udagawae* for which multiple genome assemblies were available (Fig. 5). For both *hetA* and *hetC*, the topology showed a trans-species polymorphism of two alleles, with each allele being found in all four species (Fig. 5a and 5c). The phylogeny of *hetD* showed a similar pattern of trans-species polymorphism, but instead with three alleles (Fig. 5d). Of these three alleles for *hetD*, the D2 allele was present in all four species, but *hetD1* was not recovered in *A. udagawae*, while *hetD3* was not recovered from *A. fischeri*.

For *hetB* and *hetE*, which showed a presence/absence polymorphism between our parents (Fig. 3), we reconstructed the phylogenetic relationships of the *het* alleles with the predicted coding sequence (Fig. 5b and 5e) as well as recorded the status as presence/absence (Fig. 5f). The *hetB* protease allele was present in approximately 50% (22/46) of the genome assemblies, and notably in all six *A. udagawae* assemblies. For *hetE*, a NACHT + Ankyrin repeat protein-coding sequence was found in approximately 75% of the genomes (36/46), the phylogenetic relationship of which showed two distinct protein-coding alleles, meaning three alleles including the null allele, with a trans-species polymorphism. The absence allele was found in *A. fumigatus*, *fischeri,* and *lentulus*. In a control analysis, a phylogenetic tree of the DNA repair gene *rad51* showed monophyletic groups for each species (Fig. 5f).

### Fine-scale Population Genetics of Balancing Selection in *A. fumigatus*

**Fig. 6.**
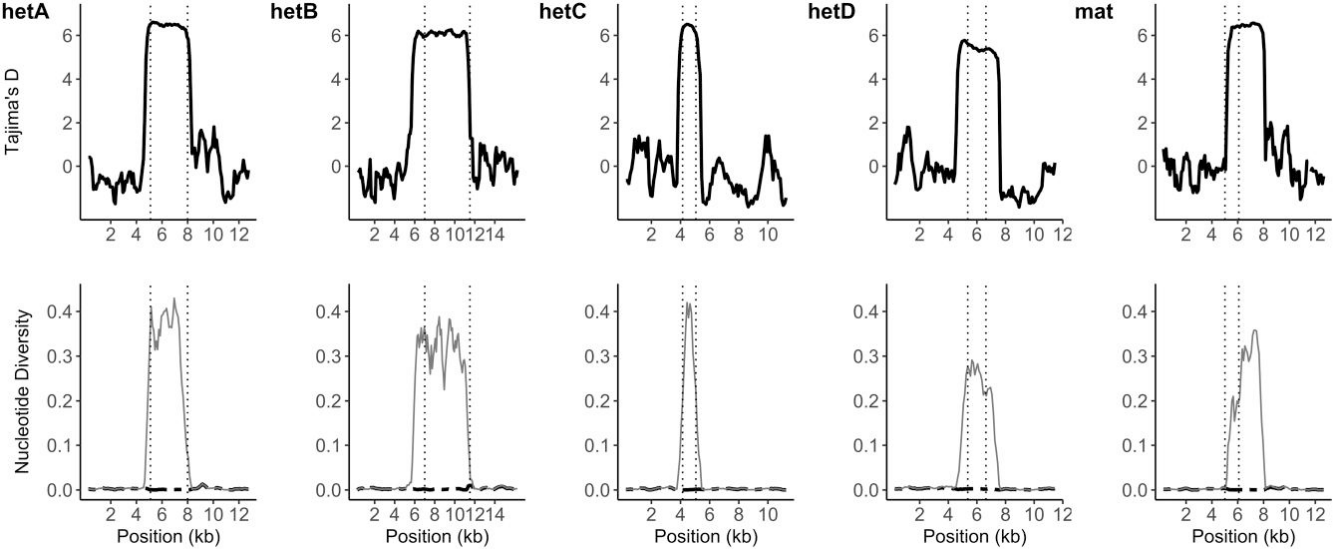
Signatures of balancing selection in *A. fumigatus* are highly restricted. Above is plotted Tajima's *D* value for the region of each of the *hetA/B/C/D* as well as the *mat* locus and 5 kb of flanking sequence. Dashed vertical lines indicate the start and stop codon of the respective gene, based on the Af293 annotation. The nucleotide diversity is plotted below, with thin lines indicating the difference between alleles, and the thick dashed line indicating nucleotide diversity within an allele.

To attempt to reconcile the lack of evidence for population-level balancing selection with the evidence of trans-species polymorphisms, we made use of public de novo genome assemblies from ∼300 previously sequenced individuals of *A. fumigatus*. Extracting the coding sequence and 5,000 bp up- and down-stream sequences allowed us to compare divergence between alleles of *hetA/B/C/D*. As a positive control, we also extracted the sequence of the *mat* locus that determines mating compatibility in this obligately outcrossing species, which is known to fall under balancing selection (May et al. 1999). Tajima's *D* value was highest across the coding sequence, reaching values greater than 6 (Fig. 6, top row). However, the drop-off to a neutral *D* value was abrupt, with values dropping below 2 within 500 bp of the start/stop codon. This abrupt and rapid change was mostly mirrored in the nucleotide diversity within and between the allele classes (Fig. 6, bottom row). An exception was found in the *mat* locus, where the nucleotide diversity was somewhat lower across the left-hand side of the coding region. Close inspection of the alignments revealed that this was due to a sharing of the terminal ∼300 bp of the α-box protein.

## Discussion

It is assumed that fusion between conspecific individuals is a risky behavior, which is rigorously policed in fungi by the heterokaryon incompatibility system. Our results here reveal the polygenic nature of this heterokaryon incompatibility in *A. fumigatus*. For the two parents used in our cross, both isolated in 2005 in Ireland, five loci contribute to their incompatibility. Using a single cross and approximately 150 offspring, we could map all responsible loci with high resolution, highlighting the power of genetics in species like *A. fumigatus* with an exceptionally high recombination rate (Auxier et al. 2023). These identified *het* genes are to our knowledge the first identified in any *Aspergillus* species and also any member of the class *Eurotiomycetes*.

Two of these *het* genes, *hetC* and *hetD*, are consistent with effector domains commonly found in NLR genes but without the other components (Uehling et al. 2017). The alleles of *hetD* encode similarly sized nucleotide phosphorylase PNP_UDP domains, while the alleles of *hetC* both encode a patatin-like domain. These patatin domains are also found in the PLP-1, which interacts with the SNARE domains of the nearby *sec*-9 gene in *N. crassa* (Heller et al. 2018). However, the action of the gene products of *hetC* and *hetD* seems to occur in isolation, since there are no neighboring genes with divergent alleles. It is interesting to note that in *N. crassa* there is another *plp* gene, *plp*-2, which also shows evidence of balancing selection, but nonallelic differences between *plp*-2 and *sec*-9 did not trigger cell death (Heller et al. 2018). As differences at a PLP appear sufficient to trigger heterokaryon incompatibility in *A. fumigatus*, perhaps there are also allelic interactions in *N. crassa plp*-2 that do not require a partner gene to trigger nonself recognition.

Alleles from two of the *het* genes we identify*, hetA* and *hetE*, have a more traditional NLR-like structure, common to fungal *het* genes as well as immune-system genes in plants and animals (Uehling et al. 2017). In our experiments, differences at *hetA* completely block heterokaryon formation, while heteroallelism at *hetE* delays, but does not prevent, heterokaryon formation. This is strikingly similar to the “partial” *het* genes previously identified in *A. nidulans* (Coenen et al. 1994). There, multiple alleles were identified that individually delayed heterokaryon compatibility; however, differences at multiple of these “partial” *het* genes was sufficient to fully block heterokaryon formation (Coenen et al. 1994). In our case, we observed delayed heterokaryon formation based on allelic differences between a coding sequence and an absence allele. It would be interesting to test the interaction between alternate alleles of *hetE* both encoding NLR proteins, and whether this interaction would also only delay or completely block heterokaryon formation. Across *A. fumigatus* and related species, we recovered two different NLR-encoding alleles for *hetE,* and one allele without a coding sequence. This situation, with two alternate protein-coding alleles and one null allele, is similar to the ABO blood group system in primates where a trans-species polymorphism exists but with null alleles private to each species (Ségurel et al. 2012). However, it is possible that the likely candidate gene, producing the NACHT + Ankyrin repeat protein, is not acting alone. Adjacent to this gene is AFUA_6G07020, an ortholog of yeast *boi1*. In yeast, this gene functions in vesicle fusion during exocytosis (Kustermann et al. 2017: 1; Masgrau et al. 2017: 1). Interestingly, a similar pairing has recently been described in the *het* genes of the plant pathogen *Botrytis cinerea*: *Bcvic1*, a NACHT + Ankyrin encoding gene, and *Bcvic2,* which encodes a SNARE syntaxin protein (Arshed et al. 2023). These SNARE domains are involved in anchoring cargoes to membranes and are also known from the *sec*-9/SNARE system in *N. crassa*, and *het-Z* of *Podospora anserina,* which both involve an NLR type protein and a SNARE protein (Heller et al. 2018). It may be that *hetE* of *A. fumigatus* has an interaction with neighboring *boi1* in an analogous role. Notably, the *sec*-9/*plp*-1 interaction in *N. crassa* has a strong phenotype at the germling stage but not in developed mycelia (Heller et al. 2018). This may point to a stronger phenotype for *het*E here, if tested during germling fusion. Recently, methods for inducing and visualizing germling fusion in *A. fumigatus* have been developed which may assist future studies (Macdonald et al. 2019).

The *hetB* locus in *A. fumigatus* presents two alleles that share no protein similarity, i.e. are idiomorphs, with one allele encoding a large protease, and the other allele a short protein without annotated domains. This large protease is predicted to contain a CHAT domain, known to function in mammalian apoptosis, and found in other metacaspases in nonmammalian lineages (Hoffman et al. 1997; Aravind and Koonin 2002; Bouchier-Hayes and Martin 2002). To our knowledge, this domain has not previously been demonstrated to be involved in heterokaryon incompatibility in any fungal lineage. However, recent work in *Neurospora* and *Podospora* has a serine protease involved in controlling gasdermin-related cell death, and genomic surveys indicated that gasdermins are often found near proteases including CHAT domains (Clavé et al. 2022). This work provides the first proof of the involvement of a CHAT protease, which may be involved with gasdermins in *A. fumigatus*. The involvement of a CHAT domain raises interesting questions about downstream pathways since fungal cell death is not considered homologous to mammalian apoptosis (Hardwick 2018; Kulkarni et al. 2019). The involvement of such domains appears to be part of an ancient defense system, with evidence that such proteins may even be involved in bacterial viral defense (Johnson et al. 2022; Wein et al. 2023). Verification of the biochemical target of the protease of *hetB*, whether a gasdermin is involved, and a potential role of the small coding sequence of the alternate allele is an important next step.

A surprising finding of this study was the lack of population-level signatures of balancing selection when calculated using window sizes common to such studies (Heller et al. 2018; Ament-Velásquez et al. 2022). It is well established that the mating locus as well as *het* genes are under negative frequency-dependent selection, leading to balanced polymorphisms. Our finding of extensive trans-species polymorphism and equal allele frequencies confirmed that balancing selection was acting as expected for these alleles. However, windowed analysis for selection using Tajima's *D* did not show strong deviations from neutral evolution. It appears this discrepancy can be reconciled through a closer analysis of genotypes. Genome-wide studies typically use blocks of 10 kb or higher to average across the genomic stochasticity of individual variants. However, this assumes that the signal from balancing selection, like any other selection, will be carried partially to the neighboring sequence, the “footprint” of selection, due to linkage with the selected locus (Hudson and Kaplan 1988; Charlesworth 2006). In *A. fumigatus*, we see that variation between allelic classes only extends several hundreds of base pairs up- or downstream of the coding regions. Such a narrow region could be related to the large population size of the species and the age of the incompatibility alleles (Charlesworth et al. 1997). This means that standard windowed analysis for this species is likely to skip over strong signals of selection, whether from balancing selection or from otherwise. Compounding this narrow footprint of balancing selection, the separation between these alleles has existed for so long that the sequences have diverged to a point that standard genomic methods fail. Within such regions, typical short-read DNA data will not be able to be mapped to the syntenic region between isolates. Downstream analysis will then generally remove such variant sites, due to “missing” data.

The balancing selection we observe on these *het* genes appears to have lasted for at least several millions of years as we find shared alleles across a group of four related species. However, we were limited in our sampling to species with multiple available genome assemblies, and thus closely related species like *A. oerlinghausenensis* could not be used (Houbraken et al. 2016), nor more distantly related species like *A. clavatus*. We would expect that the alleles for these five genes turn out to be shared across an even wider number of species, when additional genome assemblies become available. Across the species studied, we found that the allelic diversity was limited to a narrow region outside the coding region of the gene. This is likely related to the high recombination rate in this species, reducing linkage to nearby regions (Lofgren et al. 2022). This observation has been described before as “genomic islands of divergence”, but the cause was unclear at the time (Fedorova et al. 2008). Although there are few genome-wide empirical analyses of negative frequency-dependent selection, it seems that generally the window of balancing selection is wider than observed here. A recent analysis of balancing selection on the S gametic self-incompatibility locus in *Arabidopsis helleri* and *A. lyrata* showed that increased nucleotide diversity was seen several tens of kilobases from the selected locus (Veve et al. 2023). Likewise, recent analysis of the fungus *Podospora anserina* showed that signatures of balancing selection could be determined from population-level analysis looking at 10 kb windows (Ament-Velásquez et al. 2022). However, the general relationship between recombination rate and the size of the footprint is unclear, as the divergent region surrounding the *MLO2b* immunity gene in *Capsella* sp. is also tightly restricted, despite a much lower rate of recombination (Sicard et al. 2011; Koenig et al. 2019). Likewise, a narrow window of balancing selection has been observed at the mating-type locus in *N. crassa*, which has a much lower recombination rate than *A. fumigatus* (Glass et al. 1990). Clearly, the interaction between the age of balancing selection, the recombination rate, and the resulting window of sequence divergence requires future study.

We did not recover any association between the mating-type locus and heterokaryon incompatibility. In heterothallic Ascomycete species, the mating-type locus experiences balancing selection due to frequency-dependent selection. It has been previously suggested that the balancing selection arising from mating-type frequency-dependent selection may help explain the diversity of nonself recognition genes (Crozier 1986; Rousset and Roze 2007). This association is found in some fungal species such as *N. crassa*, where differences at the mating-type locus trigger heterokaryon incompatibility (Newmeyer et al. 1973). Additionally, within *Aspergilli* the mating-type locus in outcrossing *A. heterothallicus* has been genetically linked with *het-*gene activity, although the molecular genetics are unknown (Kwon and Raper 1967). However, the absence of mating-type-associated heterokaryon incompatibility has also been observed, for example in *Cryphonectria parasitica* (McGuire et al. 2004). For most species, the association remains untested. A byproduct of this absence of mating-type-associated incompatibility in *A. fumigatus* may be the formation of a sexually fertile diploid heterozygous for the mating type. This has been recently suggested in the diploid formation model of *A. latus*, although this model did not incorporate the difficulties imposed by heterokaryon incompatibility between wild isolates (Steenwyk et al. 2020). While diploids have been previously observed in clinical isolates of *A. fumigatus*, these diploids were almost completely homozygous having arisen by genome duplication, avoiding incompatibility (Engel et al. 2020). Regarding the mating-type locus itself, population-level analysis showed that the effects of balancing selection were not as sharp as expected. This is due to a partial sequence of the transcription factor of the MAT-2 allele being also found in the MAT-1 allele, as reported previously (Paoletti et al. 2005).

Of the loci described here, only for *hetE* are interactions between linked genes instead of homologs, i.e. nonallelic interactions, probable. In other species studied in detail, such nonallelic interactions seem common: *het-*R/V and *het*-C/D/E in *Podospora* (Saupe et al. 1995, 2000), the *hetC*/*pinC* and *het*-6 systems in *Neurospora* (Smith et al. 2000; Kaneko et al. 2006), the *Bcvic1/Bcvic2* system in *Botrytis*, and both the *vic2* and *vic6* loci in *Cryphonectria* (Choi et al. 2012). The extremely high recombination rate in *A. fumigatus* (1% of offspring will be nonparental across a distance of 2.5 kb) (Auxier et al. 2023) may mean that such nonallelic interaction presents a fitness cost. Any offspring with a crossover between adjacent genes in a nonallelic interaction would then contain an incompatible pair of alleles, triggering nonself-recognition within its own cytoplasm (Chevanne et al. 2009). However, even for *hetE*, which may have nonallelic interactions with *boi1*, there is a large region of high-sequence divergence, which is likely restricting recombination, as the meiotic machinery requires stretches of identical sequences during the initial homology search (Li et al. 2006). Therefore, the fact that *hetE* sits within a large divergent locus may itself prevent crossovers that would otherwise lead to self-incompatible offspring.

Fungal heterokaryon incompatibility involves a sort of paradox. It is understood to be present in all extant filamentous members of Dikaryotic fungi, yet there are no ubiquitous fungal “*het*” genes. Thus, each species, or set of related species, requires independent study to understand the repertoire of fungal *het* genes (Paoletti 2016). The five loci we identify here are unlikely to be the only variable *het* genes in the species, and analysis of additional isolates of this species likely will reveal additional *het* genes. However, detailed understanding of additional nonself recognition responses is still only an initial step in understanding heterokaryon incompatibility. The mechanism by which these allelic variants trigger cell death remains largely unknown and requires further study. Deciphering the programed cell death pathway in this species may present novel targets for treating human aspergillus infections and may open the way to eliminate incompatibility barriers to parasexual recombination for improvement of industrially important asexual *Aspergilli*.

## Materials and Methods

### Media

For heterokaryon compatibility testing, Minimal Medium (MM) was used. MM consists of 6.0 g NaNO_3_, 1.5 g KH_2_PO_4_, 0.5 g MgSO_4_. 7H_2_O, 0.5 g KCl, 10 mg of FeSO_4_, ZnSO_4_, MnCl_2_, and CuSO_4_ and 15 g agar per 1000 mL H_2_O (pH 5.8) (Zhang et al. 2019). For testing auxotrophic mutants, alternative nitrogen sources nitrate (NO_3_), nitrite (NO_2_), or hypoxanthine (C_5_H_4_N_4_O) were used at 5 mM. For routine growth, MM supplemented with urea (0.3 g/L) was used for all mutant growth and was used for general culturing of auxotrophic mutants and sexual offspring.

### Recessive Markers Introduction in the Parental Isolates

To allow for heterokaryon testing, recessive auxotrophic markers (nitrate nonutilizing mutations *nia*, *nir*, and *cnx*) were isolated from spontaneous mutations in spore suspensions of parental isolates AfIR974 and AfIR964. To isolate these spontaneous mutations, spore suspensions (approximately 1 × 10^6^ spores) were spread on MM medium supplemented with 200 mM of chlorate and 5 mM urea. As chlorate is toxic to wild-type *A. fumigatus*, this method selects for strains deficient in nitrogen metabolism. After 3 days of growth at 37°C chlorate-resistant colonies were isolated, purified, and classified as *nia*, *nir*, or *cnx* mutants based on differential N-source media tests (Cove 1976).

### Recessive Markers Segregation of Sexual Offspring

The sexual cross was made from parental isolates AfIR964*cnx* and AfIR974*nir*, which were co-inoculated on Oatmeal Agar followed by incubation at 30°C. The genotypes of the offspring were determined for a previous study (Auxier et al. 2023). Offspring were phenotyped for *cnx* and *nir* markers as above.

### Heterokaryon Compatibility Testing

Heterokaryon compatibility of offspring was tested by co-inoculating conidia (approximately 10^5^) of isolates with complementing *nir*, *nia*, or *cnx* markers on MM with NO_3_ as the sole nitrogen source (Zhang et al. 2019). All possible combinations of single mutant offspring with complementing *nir* and *cnx* markers were tested for compatibility. In addition, all *nir*, *cnx*, and *nir/cnx* offspring were tested for compatibility with *nia* mutants of parental isolates. Following co-inoculation, plates were incubated at 37°C, and heterokaryon formation, vigorous growing yet irregularly shaped colonies, was recorded after 3 and 5 days of incubation. Pairings that successfully formed heterokaryons were re-tested in triplicate, with additional testing of spore mixtures on MM + NO_3_ to ensure heterokaryotic growth was not due to contamination.

### Segregating Crosses

Segregation of individual *het* loci was tested by backcrossing offspring that differed from one of the parents at only one *het* locus, (shared alleles for *hetA*, *C*, *D*, *E* but not *hetB*, for example), and with the opposite mating type. Crosses were performed on Oatmeal Agar as above, and sexual offspring were isolated from cleistothecia following a 1 h 70°C heat-shock treatment to kill asexual spores. Only *cnx* offspring was used, 40 of which were tested for compatibility against the *nia* version of the parental isolate as above.

### *het* Loci Association Mapping

To map the regions associated with incompatibility, the 166 offspring and 2 parents could initially be split into 15 compatibility groups based on heterokaryon testing. To map the genetics of this trait, we used a set of 14,153 high-confidence markers identified previously from this cross (Auxier et al. 2023). Briefly, these markers were identified based on high-sequence quality in both parents and offspring, no evidence of heterozygosity in any parent/offspring (as all are haploid), and high mapping quality to avoid duplicated regions. Variants were called after mapping to the de novo assembly of AfIR974 parent used in this cross (Auxier et al. 2023). The genetic similarity within a group was tested using the combined Shannon Entropy of the allele frequency within a group. As each group should be defined by genetic similarity within a group for a set of loci, Shannon Entropy allows to test whether the genotypes in a group are informative for a phenotype, without the influence of the variation in compatibility group size. As the markers were all biallelic, the formula was:


$$\overset{hcg15}{\underset{hcg1}{\sum }}\;[P(a1)\times \log\;(P(a1))]+[P(a2)\times \log(P(a2))]$$

where *hcg*1 to *hcg*15 are the different heterokaryon compatibility groups, and *P*(*a*1) and *P*(*a*2) are the allele frequencies of the two alleles within each group. Thus, the association for a marker is the summed Shannon Entropy for the 15 recovered compatibility groups. To compute a null distribution, the genotypes of the compatible groupings of the 166 offspring were randomly distributed into 1,000 replicate populations with compatibility group sizes the same as our actual results. Threshold cutoff was determined on the 95% of the distribution. Fine-mapped regions were visualized with gggenes and clinkr (Gilchrist and Chooi 2021; Hackl and Ankenbrand 2022), and domains of candidate genes annotated with InterProScan (Paysan-Lafosse et al. 2022).

### *het* Gene Confirmation

To validate our predicted *het* genes as causal to the incompatible phenotype, we expressed the alternate alleles from autonomously replicating nuclear AMA1 plasmids. First, we PCR-amplified the predicted gene from gDNA of either parent using BioVeriFi polymerase (PCR Biosystems Inc, PA, USA; PB-10.42-05), including the upstream and downstream intergenic space (supplementary Data: table S5, Supplementary Material online). To include the native regulatory elements, we included approximately 750 bp of flanking regions, in some cases up to ∼50 bp of the adjacent gene. The plasmid backbone was also amplified with BioVeriFi polymerase and ligated with the insert using NEBuilder HiFi DNA Assembly Master Mix (New England Biolab Inc. #E2621L) according to manufacturer directions and transformed into Mach1 competent *E. coli* cells (ThermoFisher Scientific Inc.). Plasmid was then extracted from these cells, and ∼1 μg was used in polyethylene glycol-mediated protoplast transformation (van Rhijn et al. 2020). Transformed colonies were selected based on growth on Malt Extract Agar (20 g/L) + 300 μg/mL Hygromycin B (H0192; Duchefa Biochemie, Haarlem, the Netherlands).

### Cross-species Comparison

To assess the extent of trans-species polymorphisms, we made use of publicly available genome assemblies (supplementary File S1, Supplementary Material online). We used tblastn v2.8.1 to search the genomes of related *Aspergillus* species using predicted protein alleles from AfIR964, AfIR974, and Af293 (Camacho et al. 2009), using an *e*-value cutoff of 1*e*–75. To avoid partial matches based on conserved domain, we filtered results to be >300 bp long. The corresponding DNA sequences were extracted and aligned with mafft v7.427 using the –auto command (Katoh and Standley 2013). Phylogenetic relationships were inferred using iqtree v1.6.1 using default settings (Nguyen et al. 2015). The resulting most likely tree was midpoint rooted and visualized with ape and ggtree (Paradis et al. 2004; Yu et al. 2017).

### *A. fumigatus* Population Genome Scan

To assess the effects of balancing selection surrounding genes expected to be under balancing selection, we used data from two resequencing studies (Barber et al. 2021; Rhodes et al. 2022). From the UK dataset of Rhodes et al., we downloaded the variant file from the publication, which was mapped against the reference Af293 genome. Tajima's *D* values were calculated in windows sizes of 10,000 bp using vcftools (Danecek et al. 2011).

### *het* Gene Diversity

To determine the nucleotide diversity across the *A. fumigatus* species, we made use of genome assemblies from a recent large population resequencing study (Barber et al. 2020, 2021). The genome assemblies of the *A. fumigatus* strains used for the trans-species polymorphism analysis (see above) were added to this dataset, making a total of 334 *A. fumigatus* genome assemblies. For each of *hetA/B/C/D*, as well as the mating type as a positive control, we extracted the corresponding gene from Af293, as well as 5 kb of flanking sequence. Due to the high-sequence divergence of the *het* alleles, we used the high sequence similarity of flanking regions to isolate homologous sequences. This sequence was used with blastn to search the genomes of each assembly, and the region inside the borders of the flanking sequence was retained. The sequence was extracted using SAMtools and aligned using mafft v7.427 using the GINSI algorithm as we expected low similarity in the center of the alignment (Li et al. 2009; Katoh and Standley 2013).

## Supplementary Material

msae079_Supplementary_Data

## Data Availability

Strains and plasmids are available by contacting corresponding authors. Code and data for analyses used in this manuscript is available at: https://github.com/BenAuxier/asp_fum_het.
