# Supplementary figures and images for: The Narrow Footprint of Ancient Balancing Selection Revealed by Heterokaryon Incompatibility Genes in Aspergillus fumigatus
Source: Mol Biol Evol. 2024 Apr 23;41(5):msae079. doi: 10.1093/molbev/msae079 (PMC11138114; doi:10.1093/molbev/msae079)

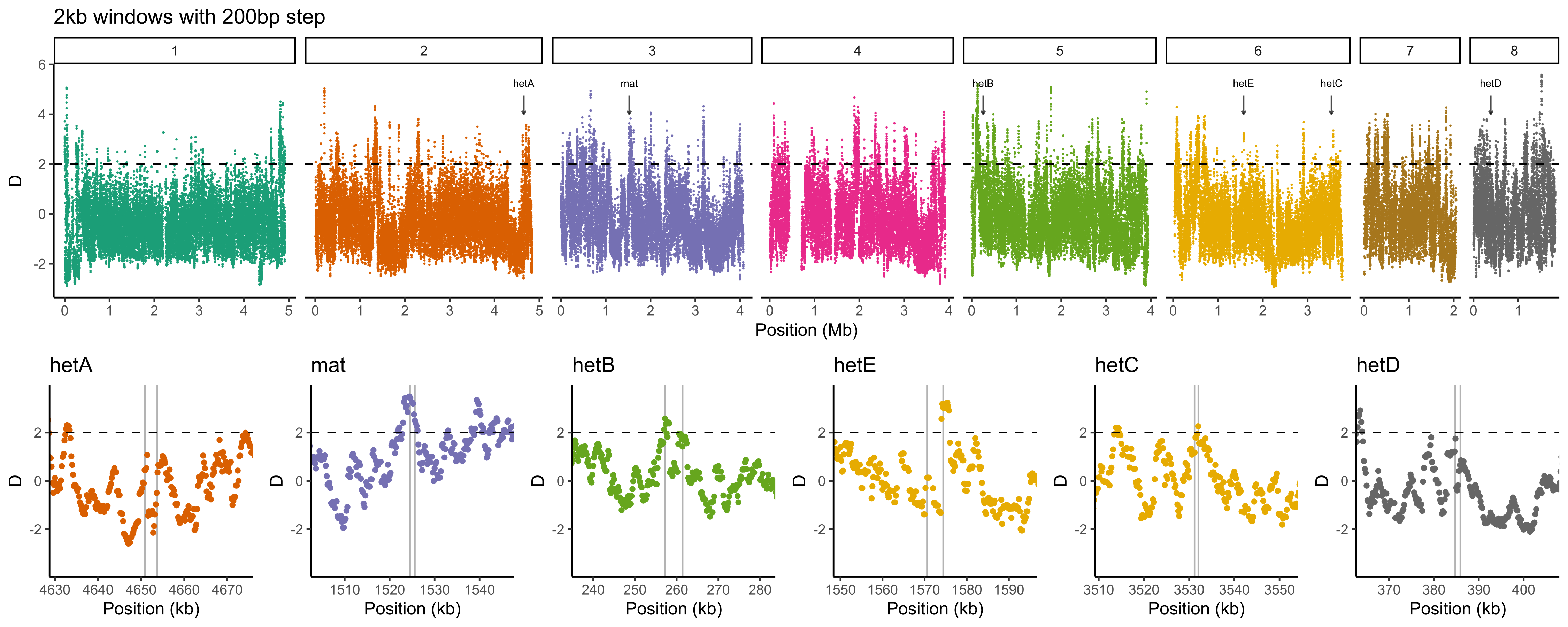

Supplement: msae079_Supplementary_Data [file msae079_supplementary_data.zip › fumigatus_2kb.png]

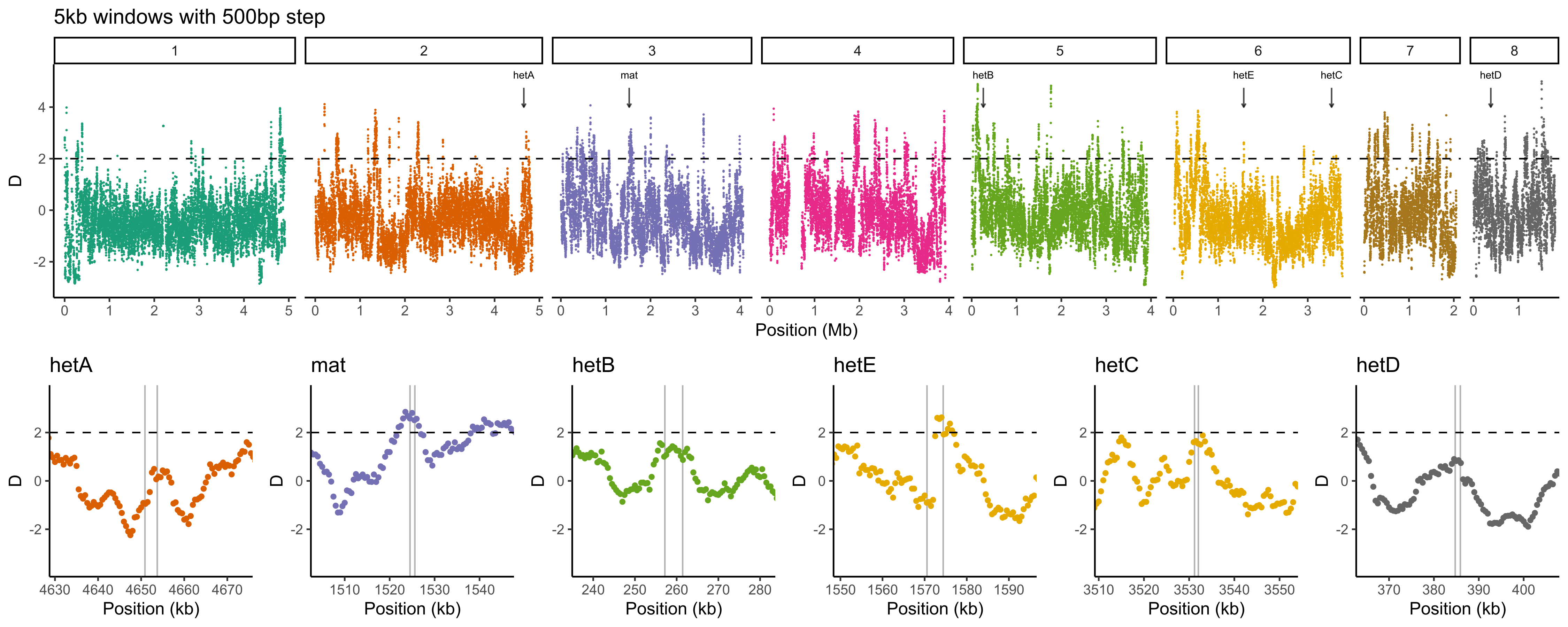

Supplement: msae079_Supplementary_Data [file msae079_supplementary_data.zip › fumigatus_5kb.png]

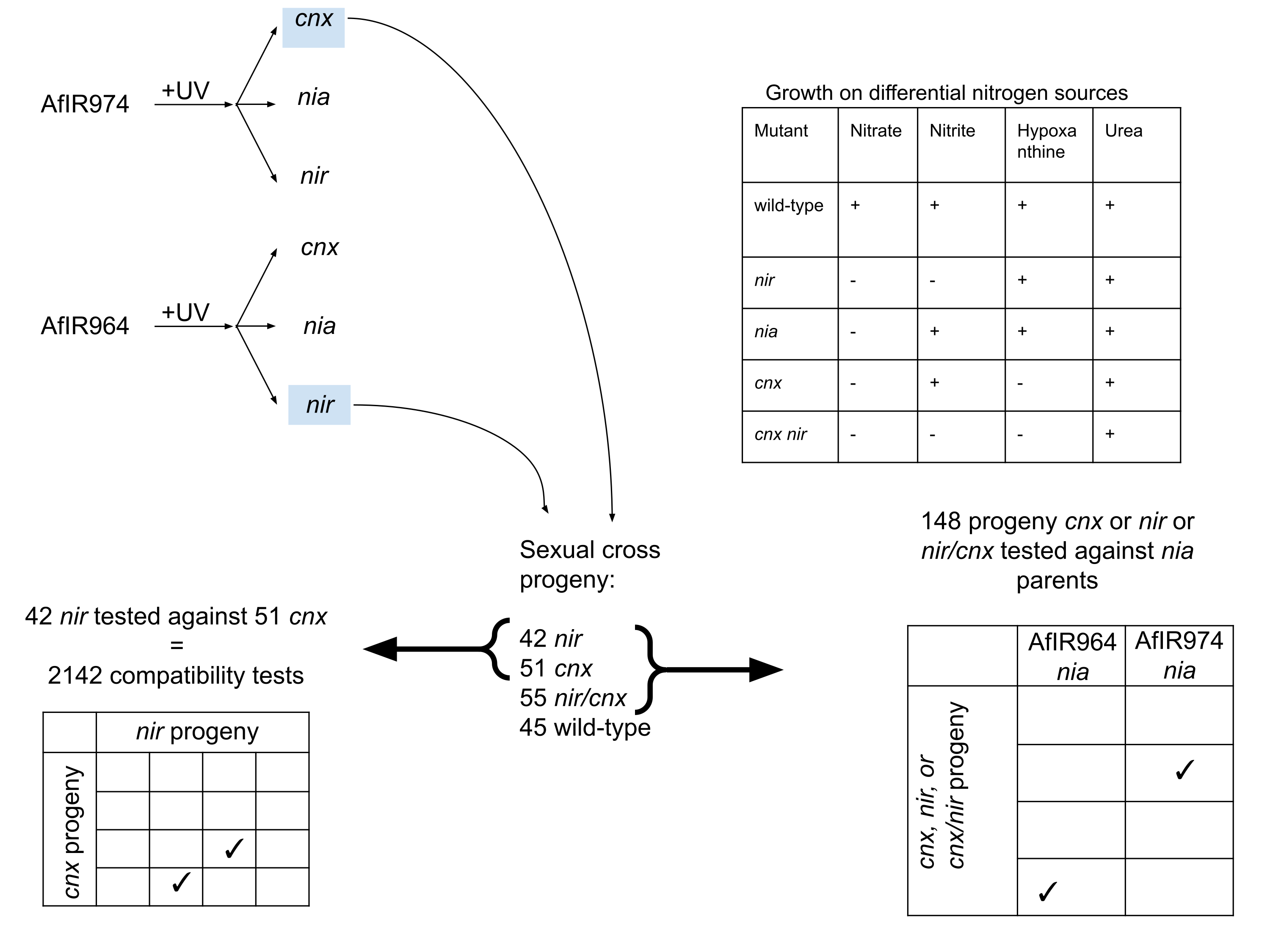

Supplement: msae079_Supplementary_Data [file msae079_supplementary_data.zip › het.testing.schema.png]

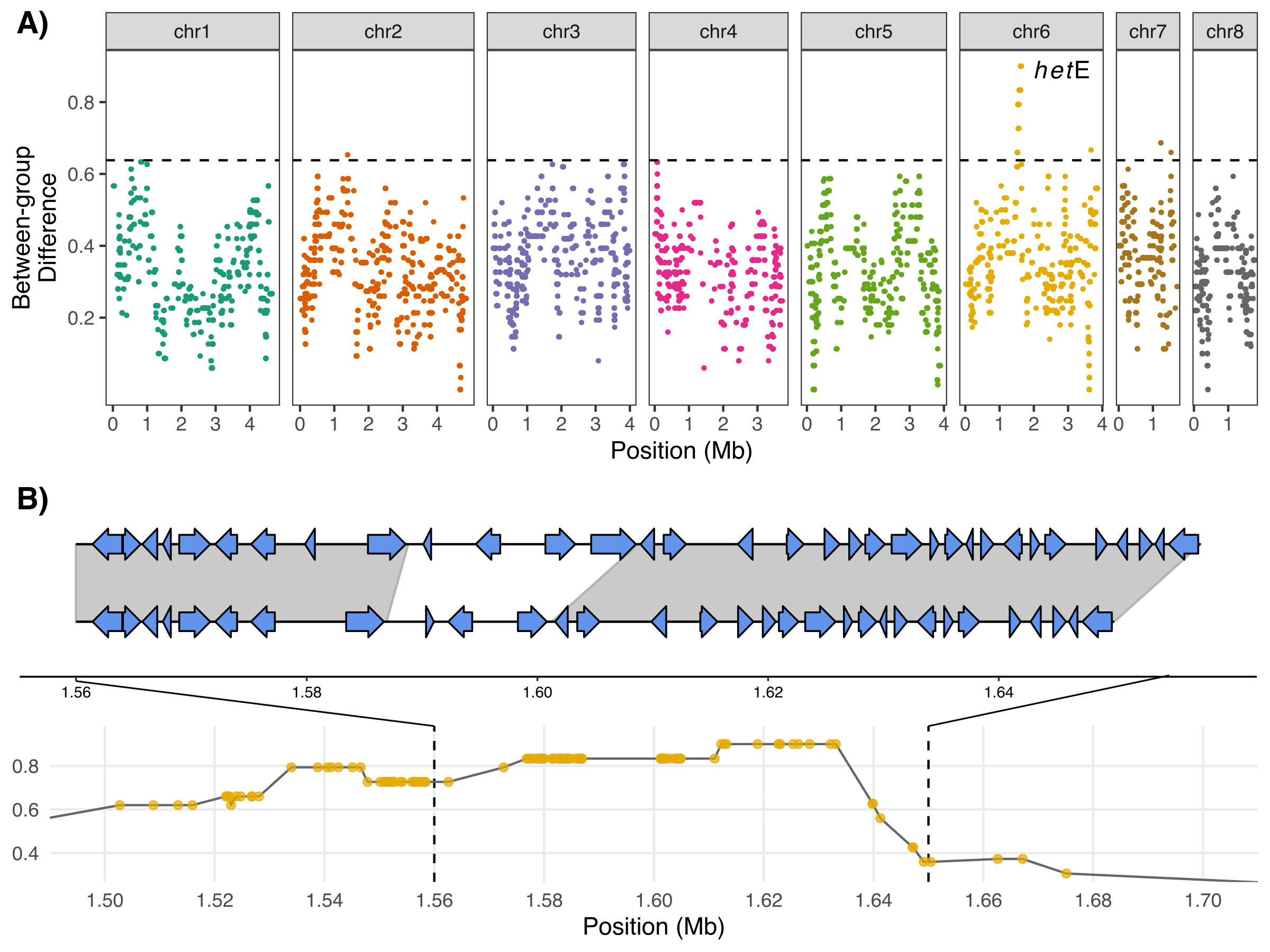

Supplement: msae079_Supplementary_Data [file msae079_supplementary_data.zip › Supplemental.hetE.2022-10-15.png]
